# Advancing knee adduction moment prediction for neuromuscular training via functional joint definitions and real–time simulation using OpenSim

**DOI:** 10.1371/journal.pone.0324985

**Published:** 2025-06-10

**Authors:** Fabian Goell, Bjoern Braunstein, Maike Stemmler, Alessandro Fasse, Dirk Abel, Kirsten Albracht

**Affiliations:** 1 Faculty of Medical Engineering and Technomathematics, Aachen University of Applied Sciences, Aachen, Germany; 2 Institute of Movement and Neurosciences, German Sport University Cologne, Cologne, Germany; 3 Institute of Biomechanics and Orthopaedics, German Sport University Cologne, Cologne, Germany; 4 German Research Centre of Elite Sport, Cologne, Germany; 5 Centre for Health and Integrative Physiology in Space (CHIPS), Cologne, Germany; 6 Institute of Automatic Control, RWTH Aachen University, Aachen, Germany; Aston University, UNITED KINGDOM OF GREAT BRITAIN AND NORTHERN IRELAND

## Abstract

Neuromuscular training to strengthen leg muscles is an important part of the treatment of musculoskeletal disorders and chronic diseases and preventing age–related muscle loss. This study evaluates different individualization approaches and their real–time implementation for OpenSim musculoskeletal models to estimate the external knee adduction moment during a leg–press exercise. A robotic neuromuscular training platform was utilized to perform isometric and dynamic leg extension exercises. Data were collected for 13 subjects using a 3D motion capture system and force plate measurements from the robotic training platform. Functional joint parameters, determined through dynamic reference movements, were integrated into the OpenSim models, allowing a personalized representation of the hip, knee, and ankle joints. This integration was compared with a conventional scaling method. The results indicate that the incorporation of functional joint axes can significantly enhance the accuracy of biomechanical simulations. These methods provide a real–time and a more precise estimate of the external knee adduction moment compared to conventional scaling approaches and underscore the importance of individualized model parameters in biomechanical research.

## 1 Introduction

Neuromuscular training to strengthen the leg muscles is an important component of the treatment for musculoskeletal disorders and chronic diseases such as knee osteoarthritis [1–4]. It is also effective for preventing age–related loss of muscle mass and strength [5–8], which is often associated with the initiation and progression of knee osteoarthritis [5,8–11]. In addition, locomotion studies have shown that knee osteoarthritis may be promoted by high forces that occur in the medial knee joint compartment [12–14]. Therefore, a robotic leg–press device was developed to manage and control exercise by simultaneously minimizing possible shear forces during neuromuscular training [15,16].

While in vivo measurement of internal forces at the medial knee joint compartment is primarily possible with invasive techniques and great effort, Kutzner *et al*. [17] showed, using in vivo measurement of medial knee joint compartment forces and an inverse dynamics (ID) approach, that the external knee adduction moment (EAM) may be a suitable surrogate for the medial knee joint compartment force during gait. Richards *et al*. [18] and Zhao *et al*. [19] came to similar conclusions with calculated instead of measured medial knee joint compartment force. Furthermore, Kolditz *et al*. [15] proved it is possible to manipulate the EAM by foot position and orientation during a leg–press exercise. To estimate the EAM, it is necessary to determine the three–dimensional (3D) force vector, and the use of a virtual human model based on a multibody system is beneficial.

Generally, for the estimation of joint moments, an integrative approach that encompasses experimental investigations and numerical multibody simulations has become an important and frequently used tool [20–22]. This approach is applied most often to the estimation of joint load during walking and running [19,22–30]. Furthermore, to obtain accurate joint angle and moment estimates, the model must represent the subject as closely as possible, and this is commonly achieved by individualizing it [31]. Thus, the most accurate approach is to use individual bone geometries of the subject derived from medical images [22]. However, this method is time consuming [22] and not always accessible. Beyond that, the model can be individualized by several different approaches to scale the lengths of the model segments together with their inertia properties to the subject using bony landmark markers or joint centers [32–38]. During the scaling process, generic joint centers and axes are adopted [20,39]. To further individualize model joints, functionally determined joint centers and axes can be used [37,40,41]; however, these were mostly utilized without the use of constraint models, although, proved to represent the joint locations more accurately [42] and result in more reliable joint angle and moment results [43]. Only recently have individual functional joint centers and axes been integrated in OpenSim models [44–46]. Because the EAM is a frontal plane moment that has only small moment arms, it is sensitive to the orientation of the adduction–abduction axis [44]. Kutzner *et al*. [17] showed, that compared to in vivo measured forces and moments, a model with functional joint axes could provide an adequate approximation of the EAM. They used the symmetrical center of rotation estimate (SCoRE) [40] to determine the hip joint center (HJC) and the symmetrical axis of rotation approach (SARA) [41] to determine the functional axis of rotation (FAR) for the knee joint flexion–extension axis and ankle joint plantarflexion–dorsiflexion axis.

Given that Meireless *et al*. [44] have demonstrated the possibility and importance of integrating functionally determined knee joint axes into OpenSim models for EAM calculations, and Kutzner *et al*. [17] have generated a suitable set of definitions for functional knee, hip, and ankle joint axes, it is vital to evaluate the combined use of both approaches and the potential advantages in a leg–press exercise scenario.

Therefore, the objective of the current study was to parameterize and evaluate an OpenSim lower extremity model with integrated functional ankle, knee, and hip joints to quantify the EAM during a leg–press exercise for neuromuscular training utilizing offline and real–time analysis.

## 2 Methodology

### 2.1 Subjects

Five male and nine female volunteers provided their informed written consent to participate in this study, which was approved by the Ethics Committee of the German Sport University, Cologne (\textnumero062/2016), where the experiment was conducted. One subject could not meet the protocol requirements during the experiment. Thus, data were analyzed for 13 subjects (age: 23.5±4.4 years, body height: 174±5.2 cm, body mass: 64±8.3 kg). The recruitment period started on 5 May 2016 and ended on 25 May 2016.

### 2.2 Study design and experimental protocol

**Fig 1:**
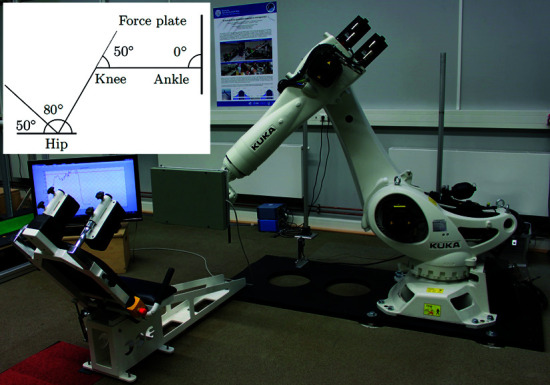
Experimental setup and schematic of the subjects initial position. Measuring setup including the KUKA KR270 2700 ultra with the AMTI OR6 Series Force Plate mounted to the end effector, the seat for the participants and the monitor for visual feedback. The schematic in the upper left corner shows the intended sagittal angles in the hip, knee, and ankle joint for the subject’s initial position with the backrest set to 70° uprise.

A robotic neuromuscular training platform (Fig 1) was developed for research purposes [15,16]. The central device was a six–axis industrial robot arm (KUKA Deutschland GmbH, Augsburg, Germany) with a 600 (H) x 400 (W) mm force plate (AMTI, Watertown, USA) mounted on the end effector and fixed to a base plate. A seat with an adjustable backrest set to 50° upward (Fig 1) and adjustable shoulder pads as counter bearings was also mounted on the base plate. This system served as a leg–press training device and provided the operator with a high degree of freedom in terms of positioning the force plate to achieve a wide range of angular settings for the lower extremity and relative foot positions.

To create the initial position for the static trials for each subject, the force plate was individually placed to obtain 80° hip joint flexion, 50° knee joint flexion, and 0° in the ankle joint.

The protocol included performing unilateral isometric leg extension exercises with the right leg in the initial (neutral) position and altered positions with 12° lateral elevation (eversion) and 12° medial elevation (inversion) of the ankle joint, which have been shown to be suitable for affecting knee joint EAM [16,47]. During each trial, the subjects applied a force for approximately 4–6 s that corresponded to 60% of the pre–recorded maximum voluntary contraction trial. To ensure that the correct amount of force was applied, visual feedback was provided on the monitor (Fig 1).

Furthermore, dynamic leg extension exercises with a constant velocity of 0.025 m/s on a linear trajectory were performed. The trajectory was set to induce a range from 80° to 40° knee flexion angle. All individual positions and trajectories were determined by an individual OpenSim model containing the robot and the subject [47]. During each dynamic trial, subjects applied the maximum force possible.

### 2.3 Data collection

**Fig 2:**
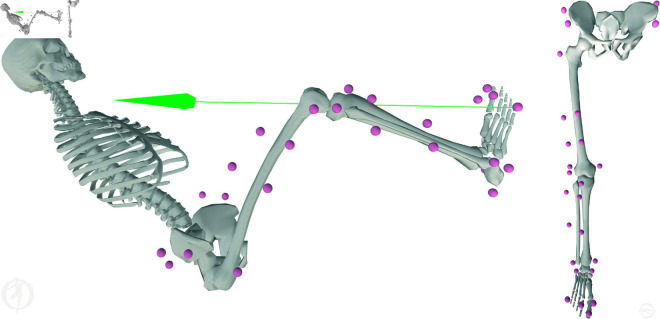
Experimental marker distribution throughout the lower limb showing landmark and cluster markers in initial pose with force (green arrow) applied (left) and in neutral standing pose (right).

The subjects’ poses and movements were recorded using a 3D motion capture system (Vicon Motion Systems Ltd, Oxford, UK), equipped with 11 infrared MX-F40 cameras operating at 100 Hz. For tracking, 32 retro–reflective spherical markers (14 mm in diameter) were attached to the torso, pelvis, and right–leg landmarks. The technical clusters, which consisted of four markers, were laterally attached to the thigh and shank segments (Fig 2). A comprehensive list of all the markers is provided in the supplementary material (S1 Table). Among the 32 retro–reflective spherical markers, 19 were used. A static reference was captured with the subjects standing erect with their feet parallel and hip–width apart and with their ankle, knee, and hip joints in the 0° positions. Dynamic references were captured, with the subjects performing a StarArc movement [48] to determine the functional HJC and an active knee and ankle movement to determine the functional knee and ankle joint axes. The posterior pelvic markers were removed after the reference recordings for the comfort of the subjects. The forces and moments applied to the robot by the subjects were recorded using the AMTI OR6 Series Force Plate (Watertown, USA) operating at 2000 Hz.

Data supporting the findings of this study are openly available under DOI: 10.6084/m9.figshare.28794887.

### 2.4 Marker data processing

The original 3D marker trajectories were automatically labeled with a predefined virtual model (Vicon Nexus 2.3, Vicon Motion Systems, Oxford, UK). Gaps, if any, were filled using the rigid body gap fill function. The posterior pelvic markers were reconstructed for each trial according to their relative position to the lateral and anterior pelvic markers in the static reference using a singular value decomposition approach [49].

### 2.5 Functional joint parameters

To reduce skin movement artifacts, the optimal common shape technique (OCST) was used [50]. The functional HJC was then calculated from the dynamic reference movement using SCoRE [40], and the FAR for the knee joint flexion–extension ($z_{knee}^{FAR}$) and the ankle joint plantarflexion–dorsiflexion ($z_{ankle}^{FAR}$) were calculated using SARA [41]. To determine the knee joint center (KJC), the midpoint of the lateral and medial femoral epicondylar markers was projected onto the functional knee axis [51]. The functional knee joint coordinate system (${JCS}_{knee}^{FAR}$) originates in the KJC. The adduction– abduction axis ($x_{knee}^{FAR}$) was then defined by the cross product of the $z_{knee}^{FAR}$ axis and the unit vector from the KJC to the HJC. The rotation axis ($y_{knee}^{FAR}$) was defined by the cross product of $z_{knee}^{FAR}$ and $x_{knee}^{FAR}$ [44]. To determine the ankle joint center (AJC), the midpoint of the lateral and medial tibial and fibular malleoli markers was projected onto the functional ankle axis [51]. The ankle joint coordinate system (${JCS}_{ankle}^{FAR}$) originates at the AJC. To obtain an orthogonal right–handed joint coordinate system, the second axis $x_{ankle}^{FAR}$ was then defined by the cross product of the $z_{ankle}^{FAR}$ and the unit vector from the AJC to the KJC. Subsequently, the third axis $y_{ankle}^{FAR}$ was defined by the cross product of $z_{ankle}^{FAR}$ and $x_{ankle}^{FAR}$. The positions of the functional joint centers and axes relative to the markers of the foot and the cluster markers of the shank and thigh, as well as the pelvic markers, were transferred from the reference trials to each isometric and dynamic leg extension trial [14].

### 2.6 Standard reference model (REF)

A model of the right lower extremity was used as the standard reference model (REF) with functional joint axes based on the SCoRE [40] and SARA [41] methods in accordance with Trepczynski *et al*. [14] and Kutzner *et al*. [17]. Its joints and segments were defined by the functional joint parameters described above. The joint angles were calculated according to Zatsiorsky *et al*. [52] and Hamill *et al*. [53]. The external joint moments were then calculated using the Newton–Euler method, applying an ID approach [54]. Joint angles and external joint moments are expressed in the distal joint segment frame.

### 2.7 Generic musculoskeletal model

The unilateral musculoskeletal model used in this study was based on the OpenSim model gait2392 [55] and adapted to meet the study’s requirements. It consisted of seven rigid body segments, which were defined by the following local reference frames: pelvis, torso, right thigh, right shank, right talus, right calcaneus, and right toes. Joints were defined by the relative motions of two joint reference frames attached to the parent and child segments, which did not necessarily coincide with the segments’ local reference frames. The model had a total of 18 degree of freedom (DOF). A joint with 6 DOF reflected the spatial motion of the pelvic segment. The torso and hip joints were modeled as ball joints with 3 DOF. The knee joint was originally modeled as a 1 DOF sliding hinge joint [55,56]. Two DOF were added in the form of two additional coordinates for knee joint adduction–abduction and rotation [57] to enable the model to output knee joint moments. Finally, the ankle, subtalar, and metatarsal joints were modeled as 1 DOF hinge joints.

The femoral segment local reference frame (${\text{LCS}}^{\text{femur}}$) originated in the HJC, located at the center of the femoral head. In the neutral position, the axes of the tibial segment reference frame (${\text{LCS}}^{\text{tibia}}$) and the talus segment reference frame (${\text{LCS}}^{\text{talus}}$) were parallel to the ${\text{LCS}}^{\text{femur}}$, with the origins at the KJC and AJC, respectively. Joints were defined by the relative motion of two reference frames - the proximal segment joint reference frame and the distal segment joint reference frame - that were embedded in the proximal or distal segment, respectively. In the generic model, they coincided with their segment reference frames. For the knee joint, the distal segment joint reference frame was ${JCS}_{knee}^{femur}$ and the proximal segment joint reference frame was ${JCS}_{knee}^{tibia}$. For the ankle joint, the distal segment joint reference frame was ${JCS}_{ankle}^{tibia}$ and the proximal segment joint reference frame was ${JCS}_{ankle}^{talus}$.

### 2.8 Scaled musculoskeletal model with conventional joint axes (Osim CON)

The Osim CON model was the generic musculoskeletal OpenSim model that was scaled using the functional AJC, KJC, and HJC to account for the individual segment lengths and their mass and inertia properties. The foot, shank cluster, and thigh cluster markers in the model were adjusted to match their respective experimental markers in the static reference.

### 2.9 Scaled musculoskeletal model with functional joint axes adjustments for the proximal segment (Osim FUN 1)

**Fig 3:**
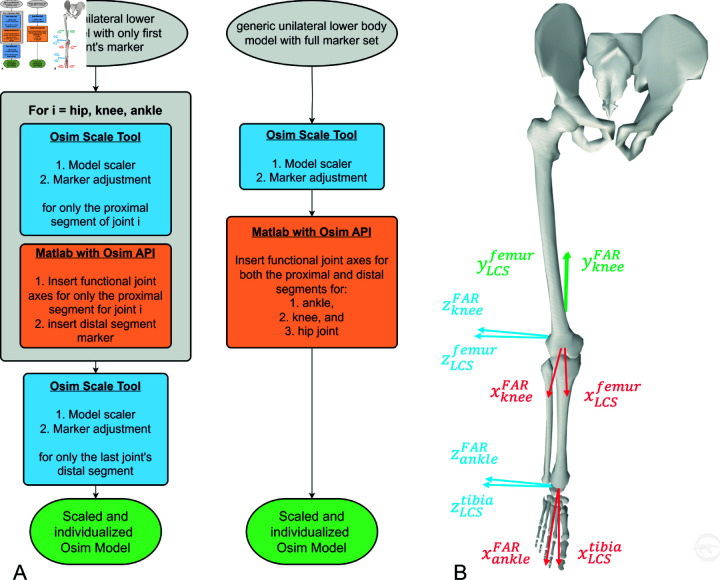
Scaling and individualization processes and resulting model. (A) The schematics describe the scaling processes for the Osim FUN 1 and Osim FUN 2 models. The processes are, respectively, embedded in the overall workflow as depicted in S1 Fig of the supplementary material. Left (Osim FUN 1): the iterative segment by segment (from proximal to distal) approach based on [44]. First, the generic OpenSim model contains only markers of the proximal segment of the first joint (hip) to be individualized. This segment was scaled and the markers were adjusted (using OpenSim scaling tool). Second, that joint’s calculated FARs were implemented (using MATLAB) and, last, the markers of that joint’s distal segment were added (using OpenSim scaling tool). The upcoming iterations of the process were performed with the next joints (knee and ankle). Right (Osim FUN 2): the all in one approach. The generic model contains all markers for all segments. The complete model was scaled and all markers were adjusted before implementing the FARs for both the proximal and distal segments of each joint. (B) OpenSim’s scaled musculoskeletal unilateral lower extremity model including the FARs and local coordinate systems (LCS) for the knee and ankle joint and the femur and tibia segment showing the axes of the functional joint coordinate systems (e.g. $x_{knee}^{FAR}$) and the axes of the respective segment coordinate systems (e.g. $x_{LCS}^{femur}$) for the joints proximal segment.

The FARs were integrated to generate the Osim FUN 1 model, adopted from Meireles *et al*. [44], and align the joint coordinate systems of the proximal segment with the coordinate system of the corresponding functional joint axes. Using an iterative approach (Fig 3A, left column), first the pelvis and thigh segments were scaled with the OpenSim scale tool including only the pelvis and thigh markers. The rotation angles to adjust the orientation of the proximal knee joint reference frame ${JCS}_{knee}^{femur}$ were calculated as rotation angles between the proximal knee joint reference frame ${JCS}_{knee}^{femur}$ and the functional knee joint coordinate system ${JCS}_{knee}^{FAR}$ that was transformed into the femur reference frame ${\text{LCS}}^{\text{femur}}$ utilizing the thigh cluster markers as common reference. In the next step, the shank segment was scaled using the OpenSim scale tool including only shank markers. The rotation angles to adjust the orientation of the proximal ankle joint reference frame ${JCS}_{ankle}^{tibia}$ were calculated as rotation angles between the proximal ankle joint reference frame ${JCS}_{ankle}^{tibia}$ and the functional ankle joint coordinate system ${JCS}_{ankle}^{FAR}$ that was transformed into the tibia reference frame ${\text{LCS}}^{\text{tibia}}$ utilizing the shank cluster markers as common reference. Finally, the foot segment was scaled with the OpenSim scale tool using only the foot markers. Markers for the distal joint segments were placed using the OpenSim scaling tool rather than inducing marker transformation using MATLAB, as performed by Meireles *et al*. [44]. Fig 3B depicts the rotated proximal joint reference frames with respect to their segment coordinate systems.

### 2.10 Scaled musculoskeletal model with functional joint axes adjustments for both adjacent segments (Osim FUN 2)

The Osim FUN 2 model with implemented FARs was obtained by integrating the functional joint reference frames into the proximal and distal joint segments using the scaled Osim CON model (Fig 3A, right column). The rotation angles to adjust the orientation of both the proximal (${JCS}_{knee}^{femur}$) and distal (${JCS}_{knee}^{tibia}$) knee joint reference frames were calculated as the rotation angles between the respective proximal (${JCS}_{knee}^{femur}$) or distal (${JCS}_{knee}^{tibia}$) knee joint reference frame and the functional knee joint coordinate system (${JCS}_{knee}^{FAR}$) that was transformed into its corresponding segment reference frame utilizing its cluster markers. The rotation angles to adjust the orientation of both the proximal (${JCS}_{ankle}^{tibia}$) and distal (${JCS}_{ankle}^{talus}$) ankle joint reference frames were calculated as the rotation angles between the respective proximal (${JCS}_{ankle}^{tibia}$) or distal (${JCS}_{ankle}^{talus}$) ankle joint reference frame and the functional ankle joint coordinate system (${JCS}_{ankle}^{FAR}$) that was transformed into its corresponding segment reference frame utilizing its cluster markers.

The Osim FUN 2 model only accounted for the differently oriented joint reference frames with respect to the segment reference frames without changing the default relative segment orientation. Whereas, the Osim FUN 1 model applying the iterative scaling and FAR integration approach allowed us to accurately monitor changes in not only the joint angle displacement but also in the inter–segment orientation in the joint reference frames.

### 2.11 Real time model (Osim RT)

For the real– time approach, a MATLAB framework was implemented using the OpenSim API [20,39,58]. The inverse kinematics (IK) and ID solver functions were executed directly for each frame with the Osim FUN 1 model. For each set of marker data, the IK solver calculated the generalized coordinates with the same optimization accuracy as the other OpenSim models. Accelerations of the generalized coordinates were obtained as the second derivative of the generalized coordinates.

For online use, the MATLAB framework was extended by incorporating the Vicon Nexus Data Stream software development kit (SDK) for real–time acquisition of 3D marker trajectories and by an interface to read the applied forces and torques of the robot control. For the purpose of this study, the marker and force data were used directly in the MATLAB framework with the respective frequency from the recorded trial data. A calculation step time of 8 ms was set as a threshold due to the plate manipulation control, which is set at a step time of 8 ms [59]. Calculations were performed with an Intel(R) Core(TM) i9-13900K CPU @ 3.00 Ghz, 32 GB RAM, and a 64-bit MS Windows OS.

### 2.12 Outcomes

For ID calculation, joint angles determined through IK were filtered with a fourth–order, zero–lag low–pass Butterworth filter with a cutoff frequency of 6 Hz, except for the Osim RT model, for which the joint angles were filtered using an exponential moving average (EMA) filter with an experimentally determined smoothing factor of 0.1525.

For the Osim RT model, the average calculation step time was determined. In addition, the number of time steps that exceeded the threshold 8 ms was given as an absolute number and as a percentage of the total number of calculated time steps.

For the static trials, the EAM was averaged over 1 s of constant force application for each of the three foot positions. Furthermore, the absolute risk of calculating an incorrect EAM with the OpenSim models compared to the REF model was determined, that is, an abduction instead of an adduction moment and vice versa. It was expressed as the number of cases of incorrectly calculated EAMs divided by the number of all the analyzed trials in percent.

Finally, a measure of fidelity was estimated in the EAM calculated with the OpenSim models. Therefore, $\Delta \;{EAM}_{footposition}$ was calculated, that is, the difference in the EAM between the two modified foot positions and the neutral foot position. It was expressed as the number of zero crossings detected of $\Delta \;{EAM}_{footposition}$ between the reference and OpenSim models divided by the number of all the analyzed trials in percent.

For dynamic trials, we extracted the mean and maximum values throughout the movement for EAM, ankle joint plantar flexion moment, knee joint flexion moment, and hip joint flexion moment.

All moments are expressed as a percentage of body weight multiplied by body height (% BWHt).

### 2.13 Sensitivity analysis

A sensitivity analysis for the OpenSim FUN 1 model was performed to evaluate the set of markers used. Eight sets of markers were defined, including or excluding specific marker groups, such as bony landmark markers, cluster markers, and additional markers, and the deviation in the knee joint adduction moment was calculated in relation to that recorded using the reference method. Marker sets S1 to S5 excluded the knee and ankle joint markers and included the cluster markers (S2) and additional markers at the hip (S3), tibia (S4), and foot (S1), or all (S5) segments. Marker sets S6 and S7 excluded the cluster markers and included the knee and ankle joint markers with (S7) or without (S6) the additional segment markers. Marker set S8 included all available markers. A comprehensive list of all the marker sets is provided in Table 1.

**Table 1:** Included markers (indicated with •) in the marker sets S1 to S8 used in the sensitivity analysis. Markers were placed at anatomical landmarks and in clusters on the pelvis and right extremity segments. The standard marker set used for the model evaluation regarding the EAM was the full marker set S8.

Segment	Anatomical landmark	S1	S2	S3	S4	S5	S6	S7	S8
Foot	Metatarsal head II	•		•	•	•	•		•
	Side of metatarsal head I	•	•	•	•	•	•	•	•
	Side of metatarsal head V	•	•	•	•	•	•	•	•
	Tip of toe II	•		•	•	•	•		•
	Heel	•	•	•	•	•	•	•	•
Shank	Medial malleolus						•	•	•
	Lateral malleolus						•	•	•
	Four–point cluster (dorsolateral)	•	•	•	•	•			•
	Caput fibulae				•	•	•		•
	Tuberositas tibiae				•	•	•		•
Thigh	Medial epicondyle						•	•	•
	Lateral epicondyle						•	•	•
	Four–point cluster (dorsolateral)	•	•	•	•	•			•
Pelvis	Anterior superior iliac spine	•	•	•	•	•	•	•	•
	Posterior superior iliac spine	•	•	•	•	•	•	•	•
	Most proximal iliac crest			•		•	•		•

### 2.14 Statistical analysis

Statistical analysis was performed using R Studio (R 4.4.0). Data were checked for normal distribution using the Shapiro–Wilk test and Q–Q plots. The variance homogeneity of the data was tested with the Levene test. Where applicable, Mauchly test for sphericity was performed. In cases of significant results in the Levene or Mauchly tests, Greenhouse–Geisser corrections were used for ANOVA.

For statistical testing of dynamic conditions for the model regarding the mean and maximum EAM and the maxima of the sagittal plane joint moments, as well as for the static condition sagittal joint flexion/dorsiflexion moments of the ankle, knee, and hip joints, independent of foot position an one–way ANOVA with repeated measures was performed. The within–subject factor was the model.

For statistical testing of static conditions for the model and foot position regarding the mean EAM, a two–factor ANOVA with repeated measures was performed. The two within–subject factors were the model and the foot position.

In any event of significance of the main effects, post hoc tests were performed with a Bonferroni–adjusted two–tailed paired t–test. If no significant differences were found between the models, Bland–Altman plots [60] were used to support the possible equality between the respective factor levels. All levels of statistical significance were set to α=.05. Data were reported as mean (M) and standard deviation (SD) and as root mean square error (RMSE) between models.

## 3 Results

### 3.1 Static conditions

The absolute risk of calculating the EAM incorrectly was quantified for the OpenSim models. There was an 8.5% risk for the Osim CON model, a 7.7% risk for the Osim FUN 1 model, and a 6.2% risk for the Osim FUN 2 model. The absolute risk of losing fidelity for $\Delta \;{EAM}_{footposition}$ was 7.7% for all three OpenSim models.

**Fig 4:**
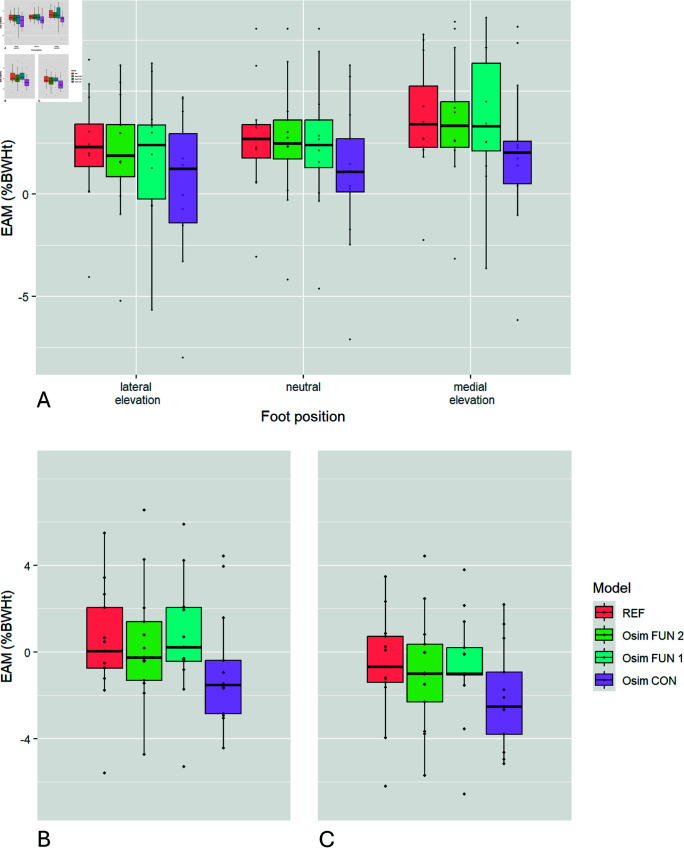
External knee adduction moment. Boxplots representing the (A) EAM values calculated for the three tested foot positions for the static trials, (B) the average and (C) the maximum EAM of the dynamic legpress trials with the REF model (red), the Osim CON model (purple), the Osim FUN 2 model (green), and the Osim FUN 1 model (blue). Black horizontal line in the box represents the median, the whiskers are defined by the 1.5*IQR (interquartile range). Black dots represent individual data points. Significant differences are stated in the text and Table 2.

The two–way ANOVA showed significant main effects for both the model and the foot position, but not for their interaction. Post hoc tests for pairwise comparison of the models showed that the EAM values calculated with the Osim FUN 1 or the Osim FUN 2 model were not significantly different from the EAM values calculated with the REF model, but from those calculated with the Osim CON model as well as the Osim CON model results differed significantly from the REF model EAM results (Fig 4A, Table 2). The RMSE values for the EAM were 2.67%BWHt for the Osim CON model, 0.90%BWHt for the Osim FUN 1 model and 0.61%BWHt for the Osim FUN 2 model, all compared to the REF model.

**Table 2:** The table presents the results of the ANOVA (last column) with the model as the main factor, and the corresponding post hoc analysis. Values are shown for the EAM in percentage of body weight times height (%BWHt) during static trials (top section), the mean EAM during dynamic trials (middle section), and the maximum EAM during dynamic trials (bottom section).

		Pairing			Diff.M (SD)	N	df	95 % CI	Test statistics	p	Cohen’s $d_{z}$	ANOVA
**STATIC**	**EAM**	REF	vs.	Osim FUN 2	–0.17 (0.60)	39	38	–0.36 to 0.02	t = –1.78	0.50	d = –0.06	${\text{F}}^{‡}$(1.34, 16.03)
		REF	vs.	Osim FUN 1	–0.28 (0.86)	39	38	–0.56 to 0.002	t = –2.01	0.31	d = –0.09	= 6.837
		**REF**	**vs.**	**Osim CON**	**–1.75 (2.05)**	**39**	**38**	**–2.41 to –1.08**	**t = –5.32**	**< 0.001**	**d = –0.55**	**p = 0.013**
		**Osim CON**	**vs.**	**Osim FUN 2**	**–1.58 (1.88)**	**39**	**38**	**–2.18 to –0.97**	**t = –5.25**	**< 0.001**	**d = –0.48**	
		**Osim CON**	**vs.**	**Osim FUN 1**	**–1.47 (2.02)**	**39**	**38**	**–2.19 to –0.75**	**t = –4.13**	**< 0.001**	**d = –0.43**	** $\eta_{p}^{2}=\mathrm{.363}$ **
		Osim FUN 2	vs.	Osim FUN 1	0.11 (0.77)	39	38	–0.15 to 0.36	t = 0.87	1	d = 0.03	
**DYNAMIC**	${EAM}_{mean}$	REF	vs.	Osim FUN 2	–0.04 (1.27)	13	12 –	0.73 to 0.81	t = 0.11	0.914	d = 0.014	${\text{F}}^{‡}$(1.45, 17.45)
		REF	vs.	Osim FUN 1	–0.30 (0.51)	13	12	–0.01 to 0.61	t = 2.13	0.054	d = 0.11	= 6.837
		**REF**	**vs.**	**Osim CON**	**–1.17 (1.84)**	**13**	**12**	**–2.28 to –0.06**	**t = –2.30**	**0.040**	**d = –0.43**	**p = 0.011**
		**Osim CON**	**vs.**	**Osim FUN 2**	**–1.21 (1.09)**	**13**	**12**	**–1.87 to –0.55**	**t = –3.99**	**0.002**	**d = –0.44**	
		**Osim CON**	**vs.**	**Osim FUN 1**	**–1.47 (1.52)**	**13**	**12**	**–2.39 to –0.56**	**t = –3.50**	**0.004**	**d = –0.54**	** $\eta_{p}^{2}=\mathrm{.364}$ **
		Osim FUN 2	vs.	Osim FUN 1	–0.26 (1.01)	13	12	–0.87 to 0.35	t = –0.94	0.365	d = –0.09	
	${EAM}_{max}$	REF	vs.	Osim FUN 2	–0.26 (1.38)	13	12	–1.09 to 0.58	t = –0.67	0.518	d = –0.10	${\text{F}}^{‡}$(1.55, 18.65)
		REF	vs.	Osim FUN 1	0.01 (0.54)	13	12	–0.32 to 0.33	t = 0.06	0.952	d = 0.004	= 6.949
		**REF**	**vs.**	**Osim CON**	**–1.42 (1.74)**	**13**	**12**	**–2.47 to –0.36**	**t = –2.93**	**0.013**	**d = –0.58**	**p = 0.009**
		**Osim CON**	**vs.**	**Osim FUN 2**	**–1.26 (1.02)**	**13**	**12**	**–1.78 to –0.54**	**t = –4.09**	**0.002**	**d = –0.46**	
		**Osim CON**	**vs.**	**Osim FUN 1**	**–1.43 (1.58)**	**13**	**12**	**–2.38 to –0.47**	**t = –3.25**	**0.007**	**d = –0.58**	** $\eta_{p}^{2}=\mathrm{.367}$ **
		Osim FUN 2	vs.	Osim FUN 1	–0.26 (1.23)	13	12	–1.01 to 0.48	t = –0.77	0.455	d = –0.10	

^†^: Repeated measures two–way ANOVA with model and foot position as within subject factors. Results in table are for factor model. Results for factor foot position are F(2,24)=15.182, *p*<.001, $\eta_{p}^{2}=\mathrm{.559}$ and for the interaction of the model and the foot position are F(1.68,20.17)=1.055, *p* = 0.355, $\eta_{p}^{2}=\mathrm{.081}$.

^‡^: Repeated measures one–way ANOVA with model as within subject factor

Further analysis using Bland–Altman plots to test the consistency and agreement of the models lacking significant differences in the EAM can be found in the supplementary material (S2 Fig).

The RMSE values for the ankle joint plantarflexion moment were 0.6%BWHt for the Osim CON model, 1.0%BWHt for the Osim FUN 1 model and 0.6%BWHt for the Osim FUN 2 model, all compared to the REF model. The RMSE values for the knee joint flexion moment were 0.8%BWHt for the Osim CON model, 0.6%BWHt for the Osim FUN 1 model, and 0.4%BWHt for the Osim FUN 2 model, all compared to the REF model. The RMSE values for the hip joint flexion moment were 3.6%BWHt for the Osim CON model, 2.0%BWHt for the Osim FUN 1 model, and 1.8%BWHt for the Osim FUN 2 model, all compared to the REF model.

The one–way ANOVA with repeated measures showed no significant main effects for the model regarding the ankle joint plantarflexion moment (F(3,48)=0.097,p=0.961,n=65), nor for the knee joint flexion moment (F(3,48)=0.022,p=0.995,n=65), or the hip joint flexion moment (F(3,48)=0.079,p=0.971,n=65).

M: Mean, SD: Standard deviation, Diff.: Deviation between the models or foot positions, RMSE: Root mean square error, N: Number of data points, df: degree of freedom, CI: Confidence interval, t: t–value, *p*: p–value, Cohen’s *d*_*z*_: Absolute effect size.

### 3.2 Dynamic conditions

The one–way ANOVA for the model showed a significant main effect for the mean EAM and for the maximum EAM. Post hoc tests for pairwise comparison of the models showed that the mean as well as the maximum EAM values calculated with the Osim FUN 1 or Osim FUN 2 model were not significantly different from the EAM values calculated with the REF model, but from those calculated with the Osim CON model as well as the results of the Osim CON model differed significantly from the REF model (Fig 4B and 4C, Table ). Further analysis using Bland–Altman plots to test the consistency and agreement of the models lacking significant differences in the EAM can be found in the supplementary material (S3 Fig).

The RMSE values for the EAM were 1.55±1.46%BWHt for the Osim CON model, 0.59±0.59%BWHt for the Osim FUN 1 model and 0.67±0.50%BWHt for the Osim FUN 2 model, all compared to the REF model.

The RMSE values for the ankle joint plantarflexion moment were 0.50 ± 0.38%BWHt for the Osim CON model, 0.70 ± 0.63%BWHt for the Osim FUN 1 model and 0.42 ± 0.29%BWHt for the Osim FUN 2 model, all compared to the REF model. The RMSE values for the knee joint flexion moment were 0.81 ± 0.76%BWHt for the Osim CON model, 0.64±0.41%BWHt for the Osim FUN 1 model, and 0.64±0.46%BWHt for the Osim FUN 2 model, all compared to the REF model. The RMSE values for the hip joint flexion moment were 2.77±3.75%BWHt for the Osim CON model, 2.04±1.68%BWHt for the Osim FUN 1 model, and 2.53±3.71%BWHt for the Osim FUN 2 model, all compared to the REF model.

The one–way ANOVA with repeated measures did not show a significant main effect for the model for the ankle joint plantarflexion moment (F(3,48)=0.064,p=0.979,n=52), the knee joint flexion moment (F(3,48)=0.029,p=0.993,n=52), nor the hip joint flexion moment (F(3,48)=0.295,p=0.829,n=52). A boxplot representation of the data can be found in the supplementary material (S4 Fig).

### 3.3 Sensitivity analysis - Marker set

The sensitivity analysis revealed that the marker sets S6 and S7, which included the lateral and medial epicondyle and the lateral and medial malleolus markers and excluded the cluster markers, were associated with the greatest deviations. Using the S5 and S8 marker sets resulted in the smallest deviations in the EAM values between the Osim FUN 1 and REF models (Table 3).

**Table 3:** RMSE of the difference, mean absolute difference, and maximum absolute difference of the EAM between the Osim FUN 2 model using the marker sets S1 to S8 and the REF model calculated for the dynamic trials.

	S1	S2	S3	S4	S5	S6	S7	S8
RMSE	0.60	0.61	0.59	0.60	0.59	0.80	0.99	0.57
Mean	0.52	0.52	0.52	0.51	0.51	0.72	0.84	0.49
Max	1.45	1.58	1.37	1.37	1.27	3.30	3.06	1.28

All values are given in percentage of body weight times height (%BWHt). RMSE: Root mean square error, Mean: Mean absolute difference, Max: Maximum absolute difference.

### 3.4 Real time simulation

**Fig 5:**
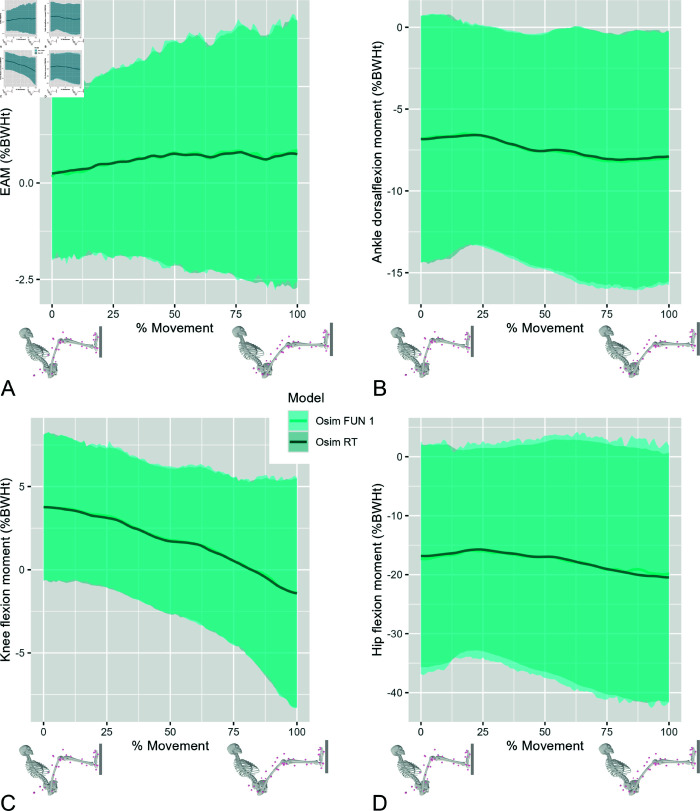
Osim RT vs Osim FUN 1 joint moments. The EAM (A), the ankle joint dorsiflexion moment (B), the knee joint flexion moment (C), and the hip joint flexion moment (D) as functions of the normalized leg extension movement calculated with the Osim FUN 1 (blue) and the Osim RT model (dark blue). The lines represent the respective average moments, and the accordingly colored areas represent their standard deviation. The visualization of the Osim model represent the start and end position at 0% and 100% of the leg extension movement, respectively.

The Osim RT FUN 1 model was calculated with a step time of 5.4 ± 0.4ms with a maximum of 21.1ms. An average of 3.44 ± 0.8 (5.4%) calculation steps exceeded the threshold of 8ms. The RMSE value for the EAM was 0.11 ± 0.04%BWHt, for the ankle joint dorsiflexion moment it was 0.15 ± 0.05%BWHt, for the knee joint flexion moment it was 0.17 ± 0.05%BWHt, and for the hip joint flexion moment it was 0.57 ± 0.12%BWHt. Figure 5 shows the joint moments as functions of normalized leg extension movement.

## 4 Discussion

The objective of the present study was to integrate functional hip, knee, and ankle joints into a unilateral lower extremity OpenSim model and parameterize the model to determine the EAM within isometric and dynamic leg–press exercise scenarios. Two approaches were applied to integrate the functional joints. The resultant models, together with an OpenSim model without functional joints, were compared to a reference model with functional joints but without utilizing OpenSim. Furthermore, a real–time approach using the Osim FUN 1 model was tested. With a focus on determining the EAM, we have shown that it is possible to approximate the results of the reference model using OpenSim models with the same functional hip, knee and ankle joint centers and the same functional knee and ankle joint axes.

Our results indicate that the Osim FUN 1 and the Osim FUN 2 models are well parameterized models for determining the EAM in the tested isometric and dynamic leg–press exercise scenarios. Without differing from the REF model statistically significant, and with a low risk of calculating the EAM incorrectly (6.2% and 7.7% respectively) or of losing fidelity for $\Delta \;{EAM}_{footposition}$ (both 7.7%), these models have proven to be suitable.

The results generated using the Osim CON model differed significantly from those generated using the REF model for dynamic and isometric conditions, a finding that aligns with the results of Meireles *et al*. [44]. Hence, the definitions of the joint coordinate systems are essential for the calculation of the EAM, and different definitions of the joint coordinate system lead to different EAM results.

In addition, our results show that the adapted FAR–integration approach (Osim FUN 1), which was based on the novel method for integrating functionally defined knee joint axes into OpenSim [44], is an effective method. Notably, because the markers of the distal joint segment need positional adjustment after integration of the joint’s FAR, when this method is used with multiple functionally determined joints, the scaling and FAR integration must be conducted in an iterative manner. We also found that the FAR–integration approach that involved adjusting the joint reference frames of both adjacent segments (Osim FUN 2) is not inferior for the determination of the EAM. Since no marker adjustments are needed in this approach, the scaling process can be performed for all segments at once. We managed to adopt the advantages of using functional joint centers and axes as shown in several previous studies [42] and integrated them into OpenSim models. Only a few studies showed that this was done before. Werling *et al*. [45] and Hammond *et al*. [46] developed optimization pipelines that use not only static and dynamic reference recordings, but also the results of the inverse kinematics of the recordings to be examined. Our approach, which is concise with Meireless *et al*. [44], is based on the direct editing of the joint axes in the model file. Although reportedly less accurate than model individualization using medical imaging [22], a motion capture based method was presented that relies on static and dynamic reference recordings.

The sagittal plane joint moments calculated with the OpenSim models did not differ significantly from those calculated with the REF model, regardless of the different joint definitions. This finding supports the common assertion that OpenSim models are robust and valid, especially for sagittal plane joint parameter analysis [19,23–30]. Thus, our results suggest that the lower extremity OpenSim model did not lose accuracy and reliability when equipped with functionally determined joints.

The Osim RT model reproduced the results of the Osim FUN 1 model almost identically with deviation of the EAM smaller than 0.11%BWHt and of the ankle joint dorsiflexion, knee joint flexion, and hip joint flexion moments smaller than 0.15%, 0.17% and 0.57%BWHt, respectively. Small deviation may occur due to different filter methods for joint angles and due to double differentiation of the joint angles frame–by–frame to gain angular accelerations. In addition, to compensate for the inability to fill gaps in the marker data during online real–time simulation, existing Kalman filter approaches [61,62], for example, should be used to ensure the completeness of the marker data.

The sensitivity analysis revealed, that the selected markers affected the EAM results and showed that a well distributed marker set is favorable, which is consistent with the results of Keizer *et al*. [63]. The marker sets that excluded the thigh and shank cluster markers (S6 and S7, Table 1) showed the greatest RMSE and mean deviation from the values recorded using the REF model (Table 3). This finding implies that it is essential to use these cluster markers to minimize deviation from the REF model. The marker set S8, which included the thigh and shank cluster markers and the femoral epicondyle and tibial/fibular malleoli markers, showed the smallest RMSE and mean deviation from the values recorded using the REF model. Including the landmark markers that had low weights compared to the cluster markers was helpful but not necessary; the use of the marker sets that had cluster markers but not landmark markers resulted in only slightly larger RMSE (<0.04%BWHt) and mean deviation (<0.04%BWHt) values (Table 3).

We used two FAR–integration methods and a standard scaling process to generate two individualized OpenSim models (Osim FUN 1 and FUN 2) that were shown to be suitable for frontal plane joint moment analysis. This also holds if the models are integrated in a frame–by–frame real–time approach. Thus, our approaches allow online monitoring of additional parameters during leg–press exercises beyond external parameters such as force and pose or position. Initially developed for rehabilitative and preventive neuromuscular training supporting to achieve sufficient training stimuli while reducing unwanted stress [64,65] in a robotic leg–press exercise [66,67], further conceivable application fields could be highly specific training in elite sports or real–time load monitoring in industrial ergonomics. Human–robot interaction in general could benefit from model– based real–time motion and load monitoring in addition to post hoc processing of pre–recorded tasks [68]. The model approach presented using functional joint parameters allows calculation of external joint moments. At this stage, the aim was not to map the compression forces of the medial compartment in the knee joint but rather to make the model more realistic. For this reason, the REF model was chosen based on Kutzner *et al*. [17]. To the authors’ knowledge, their study is the only study to combine EAM results of a model with functional joint axes and directly measured compression forces in implant supplied knee joints. To overcome the mentioned limitation, the model would benefit from further development (Fig 3 in [69]): Extension of the individualization of the model by medical imaging that provides individual bone and muscle geometry, additional use of EMG signals to provide muscle activity, and extension of the model by tissue and mechanobiological components and neuromusculoskeletal simulations. However, there are challenges for practical integration into robotic or automated training devices. First, sufficient computing power must be available. For possible real–time visualization, sufficient graphical processing power must also be available. In addition, suitable physical and software interfaces must be created to transfer the streaming data seamlessly and at sufficient speed. Depending on the given or applicable infrastructure, this could be achieved using UDP or TCP/IP connections between the robot controller and the computer that runs the real–time model on a local network specifically created for the robotic training device. Furthermore, local network servers could be used, for example an OPC server, which is accessible to every system component. Landmark markers at the knee and ankle joints should only be used in combination with segment cluster markers; therefore, there is some leeway in how many markers are included in the calculation.

In conclusion, frontal plane joint moments, such as the EAM, should best be analyzed using models that include functionally determined joint axes. In this study, two approaches were used to integrate functionally determined joint centers and axes into a scaled unilateral lower extremity OpenSim model for the hip, knee, and ankle joints: an approach adopted from a previous study [44] and a novel approach. This advancement in predicting EAM could help improve monitor neuromuscular training by enabling the observation of the EAM and thus enables manipulation of the EAM with respective training devices. Furthermore, the presented methodology of functional joint center integration could be a valuable addition to the use of the existing standard joint used in existing models, depending on the intended use. In addition, we have shown that these highly individualized OpenSim models can be very accessible computed in parallel with exercise performance using the OpenSim API in Matlab for online monitoring and control loop integration of a respective training device, and thus allowing a step towards automated training.

## Supporting information

S1 Table Comprehensive list of all markers used and their location (PDF)

S1 Fig Workflow schematic of the overall pre- and post-processing approach. C3d files containing 3D marker trajectories and force data (green) are pre-processed. The generic OpenSim model is scaled according to the individualized model variations which is described in detail in Sects 2.9 and 2.10 and displayed in Fig 3A.
(PDF)

S2 Fig Bland–Altman plot for the comparison of the EAM calculated from the static trials with the REF and the Osim FUN 1 model (a), calculated with the REF and the Osim FUN 2 model (b), and calculated with the Osim FUN 1 and the Osim FUN 2 model. (PDF)

S3 Fig Bland–Altman plot for the comparison of the mean EAM calculated from the dynamic trials with the REF and the Osim FUN 1 model (a), calculated with the REF and the Osim FUN 2 model (b), and calculated with the Osim FUN 1 and the Osim FUN 2 model (c) and for the comparison of the maximum EAM calculated from the dynamic trials with the REF and the Osim FUN 1 model (d), calculated with the REF and the Osim FUN 2 model (e), and calculated with the Osim FUN 1 and the Osim FUN 2 model (f). (PDF)

S4 Fig Boxplots representing the maximum ankle joint dorsiflexion, knee joint flexion and the hip joint flexion moment of the dynamic legpress trials calculated with the REF model (red), the Osim CON model (purple), the Osim FUN 2 model (green), and the Osim FUN 1 model (blue). (PDF)

## Abbreviations

AJC: Ankle joint center
DOF: Degree of freedom
EAM: External knee adduction moment
EMA: Exponential moving average
FAR: Functional axis of rotation
HJC: Hip joint center
ID: Inverse dynamics
IK: Inverse kinematics
JCS: Joint coordinate system
KJC: Knee joint center
LCS: Local coordinate system
OCST: Optimal common shape technique
RMSE: Root mean square error
SPM: Statistical parameter mapping
SARA: Symmetrical axis of rotation approach
SCoRE: Symmetrical center of rotation estimate
SDK: Software development kit

## References

[1] Pedersen BK, Saltin B. Evidence for prescribing exercise as therapy in chronic disease. Scand J Med Sci Sports. 2006;16 Suppl 1:3–63. doi: 10.1111/j.1600-0838.2006.00520.x 16451303

[2] Maestroni L, Read P, Bishop C, Papadopoulos K, Suchomel TJ, Comfort P, et al. The benefits of strength training on musculoskeletal system health: practical applications for interdisciplinary care. Sports Med. 2020;50(8):1431–50. doi: 10.1007/s40279-020-01309-5 32564299

[3] Lim J, Choi A, Kim B. The effects of resistance training on pain, strength, and function in osteoarthritis: systematic review and meta-analysis. J Pers Med. 2024;14(12):1130. doi: 10.3390/jpm14121130 39728043 PMC11676110

[4] Ding X, Yang Y, Xing Y, Jia Q, Liu Q, Zhang J. Efficacy of lower limb strengthening exercises based on different muscle contraction characteristics for knee osteoarthritis: a systematic review and network meta-analysis. Front Med (Lausanne). 2024;11:1442683. doi: 10.3389/fmed.2024.1442683 39386751 PMC11461219

[5] Narici MV, Maffulli N. Sarcopenia: characteristics, mechanisms and functional significance. Br Med Bull. 2010;95:139–59. doi: 10.1093/bmb/ldq008 20200012

[6] Karamanidis K, Oberländer KD, Niehoff A, Epro G, Brüggemann G-P. Effect of exercise-induced enhancement of the leg-extensor muscle-tendon unit capacities on ambulatory mechanics and knee osteoarthritis markers in the elderly. PLoS One. 2014;9(6):e99330. doi: 10.1371/journal.pone.0099330 24905024 PMC4048280

[7] McAlindon TE, Bannuru RR, Sullivan MC, Arden NK, Berenbaum F, Bierma-Zeinstra SM, et al. OARSI guidelines for the non-surgical management of knee osteoarthritis. Osteoarthritis Cartilage. 2014;22(3):363–88. doi: 10.1016/j.joca.2014.01.003 24462672

[8] Seene T, Kaasik P, Seppet E. Changes in myofibrillar and mitochondrial compartments during increased activity: dependance from oxidative capacity of muscle. Health. 2017;09(05):779–98. doi: 10.4236/health.2017.95056

[9] Janssen I, Heymsfield SB, Ross R. Low relative skeletal muscle mass (sarcopenia) in older persons is associated with functional impairment and physical disability. J Am Geriatr Soc. 2002;50(5):889–96. doi: 10.1046/j.1532-5415.2002.50216.x 12028177

[10] Mian OS, Baltzopoulos V, Minetti AE, Narici MV. The impact of physical training on locomotor function in older people. Sports Med. 2007;37(8):683–701. doi: 10.2165/00007256-200737080-00003 17645371

[11] Piasecki M, Ireland A, Jones DA, McPhee JS. Age-dependent motor unit remodelling in human limb muscles. Biogerontology. 2016;17(3):485–96. doi: 10.1007/s10522-015-9627-3 26667009 PMC4889636

[12] Andriacchi TP, Mündermann A, Smith RL, Alexander EJ, Dyrby CO, Koo S. A framework for the in vivo pathomechanics of osteoarthritis at the knee. Ann Biomed Eng. 2004;32(3):447–57. doi: 10.1023/b:abme.0000017541.82498.37 15095819

[13] Reeves ND, Bowling FL. Conservative biomechanical strategies for knee osteoarthritis. Nat Rev Rheumatol. 2011;7(2):113–22. doi: 10.1038/nrrheum.2010.212 21289615

[14] Trepczynski A, Kutzner I, Kornaropoulos E, Taylor WR, Duda GN, Bergmann G, et al. Patellofemoral joint contact forces during activities with high knee flexion. J Orthop Res. 2012;30(3):408–15. doi: 10.1002/jor.21540 22267190

[15] Kolditz M, Albin T, Brüggemann G-P, Abel D, Albracht K. Robotergestütztes System für ein verbessertes neuromuskuläres Aufbautraining der Beinstrecker. at - Automatisierungstechnik. 2016;64(11):905–14. doi: 10.1515/auto-2016-0044

[16] Kolditz M, Albin T, Albracht K, Bruggemann G-P, Abel D. Isokinematic leg extension training with an industrial robot. In: 2016 6th IEEE International Conference on Biomedical Robotics and Biomechatronics (BioRob). 2016. p. 950–5. doi: 10.1109/biorob.2016.7523750

[17] Kutzner I, Trepczynski A, Heller MO, Bergmann G. Knee adduction moment and medial contact force–facts about their correlation during gait. PLoS One. 2013;8(12):e81036. doi: 10.1371/journal.pone.0081036 24312522 PMC3847086

[18] Richards RE, Andersen MS, Harlaar J, van den Noort JC. Relationship between knee joint contact forces and external knee joint moments in patients with medial knee osteoarthritis: effects of gait modifications. Osteoarthritis Cartilage. 2018;26(9):1203–14. doi: 10.1016/j.joca.2018.04.011 29715509

[19] Zhao D, Banks SA, Mitchell KH, D’Lima DD, Colwell CW Jr, Fregly BJ. Correlation between the knee adduction torque and medial contact force for a variety of gait patterns. J Orthop Res. 2007;25(6):789–97. doi: 10.1002/jor.20379 17343285

[20] Delp SL, Anderson FC, Arnold AS, Loan P, Habib A, John CT, et al. OpenSim: open-source software to create and analyze dynamic simulations of movement. IEEE Trans Biomed Eng. 2007;54(11):1940–50. doi: 10.1109/TBME.2007.901024 18018689

[21] Pandy MG, Andriacchi TP. Muscle and joint function in human locomotion. Annu Rev Biomed Eng. 2010;12:401–33. doi: 10.1146/annurev-bioeng-070909-105259 20617942

[22] Abdullah M, Hulleck AA, Katmah R, Khalaf K, El-Rich M. Multibody dynamics-based musculoskeletal modeling for gait analysis: a systematic review. J Neuroeng Rehabil. 2024;21(1):178. doi: 10.1186/s12984-024-01458-y 39369227 PMC11452939

[23] Besier TF, Lloyd DG, Ackland TR, Cochrane JL. Anticipatory effects on knee joint loading during running and cutting maneuvers. Med Sci Sports Exerc. 2001;33(7):1176–81. doi: 10.1097/00005768-200107000-00015 11445765

[24] Lloyd DG, Besier TF. An EMG-driven musculoskeletal model to estimate muscle forces and knee joint moments in vivo. J Biomech. 2003;36(6):765–76. doi: 10.1016/s0021-9290(03)00010-1 12742444

[25] Walter JP, D’Lima DD, Colwell CW Jr, Fregly BJ. Decreased knee adduction moment does not guarantee decreased medial contact force during gait. J Orthop Res. 2010;28(10):1348–54. doi: 10.1002/jor.21142 20839320 PMC2984615

[26] Fregly BJ, Besier TF, Lloyd DG, Delp SL, Banks SA, Pandy MG, et al. Grand challenge competition to predict in vivo knee loads. J Orthop Res. 2012;30(4):503–13. doi: 10.1002/jor.22023 22161745 PMC4067494

[27] Shull PB, Shultz R, Silder A, Dragoo JL, Besier TF, Cutkosky MR, et al. Toe-in gait reduces the first peak knee adduction moment in patients with medial compartment knee osteoarthritis. J Biomech. 2013;46(1):122–8. doi: 10.1016/j.jbiomech.2012.10.019 23146322

[28] Lerner ZF, DeMers MS, Delp SL, Browning RC. How tibiofemoral alignment and contact locations affect predictions of medial and lateral tibiofemoral contact forces. J Biomech. 2015;48(4):644–50. doi: 10.1016/j.jbiomech.2014.12.049 25595425 PMC4330122

[29] Johnson WR, Mian A, Lloyd DG, Alderson JA. On-field player workload exposure and knee injury risk monitoring via deep learning. J Biomech. 2019;93:185–93. doi: 10.1016/j.jbiomech.2019.07.002 31307769

[30] Lenton G, Doyle T, Lloyd D, Pizzolato C, Saxby D. Hip joint contact forces increase in response to greater body-borne loads and faster walking speeds. 2019. p. 1047. https://www.isb2019.com/

[31] Lund ME, Andersen MS, de Zee M, Rasmussen J. Scaling of musculoskeletal models from static and dynamic trials. Int Biomech. 2015;2(1):1–11. doi: 10.1080/23335432.2014.993706

[32] Inman VT. The joints of the ankle. Baltimore: Williams & Wilkins. 1976.

[33] Bell AL, Pedersen DR, Brand RA. A comparison of the accuracy of several hip center location prediction methods. J Biomech. 1990;23(6):617–21. doi: 10.1016/0021-9290(90)90054-7 2341423

[34] Vaughan CL, Davis BL, O’Connor JC. Dynamics of human gait. Human Kinetics Publishers. 1992.

[35] Churchill DL, Incavo SJ, Johnson CC, Beynnon BD. The transepicondylar axis approximates the optimal flexion axis of the knee. Clin Orthop Relat Res. 1998;(356):111–8. doi: 10.1097/00003086-199811000-00016 9917674

[36] Leardini A, Cappozzo A, Catani F, Toksvig-Larsen S, Petitto A, Sforza V, et al. Validation of a functional method for the estimation of hip joint centre location. J Biomech. 1999;32(1):99–103. doi: 10.1016/s0021-9290(98)00148-1 10050957

[37] Besier TF, Sturnieks DL, Alderson JA, Lloyd DG. Repeatability of gait data using a functional hip joint centre and a mean helical knee axis. J Biomech. 2003;36(8):1159–68. doi: 10.1016/s0021-9290(03)00087-3 12831742

[38] Bakke D, Besier T. Shape model constrained scaling improves repeatability of gait data. J Biomech. 2020;107:109838. doi: 10.1016/j.jbiomech.2020.109838 32517858

[39] Seth A, Hicks JL, Uchida TK, Habib A, Dembia CL, Dunne JJ, et al. OpenSim: Simulating musculoskeletal dynamics and neuromuscular control to study human and animal movement. PLoS Comput Biol. 2018;14(7):e1006223. doi: 10.1371/journal.pcbi.1006223 30048444 PMC6061994

[40] Ehrig RM, Taylor WR, Duda GN, Heller MO. A survey of formal methods for determining the centre of rotation of ball joints. J Biomech. 2006;39(15):2798–809. doi: 10.1016/j.jbiomech.2005.10.002 16293257

[41] Ehrig RM, Taylor WR, Duda GN, Heller MO. A survey of formal methods for determining functional joint axes. J Biomech. 2007;40(10):2150–7. doi: 10.1016/j.jbiomech.2006.10.026 17169365

[42] Fiorentino NM, Kutschke MJ, Atkins PR, Foreman KB, Kapron AL, Anderson AE. Accuracy of functional and predictive methods to calculate the hip joint center in young non-pathologic asymptomatic adults with dual fluoroscopy as a reference standard. Ann Biomed Eng. 2016;44(7):2168–80. doi: 10.1007/s10439-015-1522-1 26645080 PMC4893978

[43] Reinbolt JA, Schutte JF, Fregly BJ, Koh BI, Haftka RT, George AD, et al. Determination of patient-specific multi-joint kinematic models through two-level optimization. J Biomech. 2005;38(3):621–6. doi: 10.1016/j.jbiomech.2004.03.031 15652563

[44] Meireles S, De Groote F, Van Rossom S, Verschueren S, Jonkers I. Differences in knee adduction moment between healthy subjects and patients with osteoarthritis depend on the knee axis definition. Gait Posture. 2017;53:104–9. doi: 10.1016/j.gaitpost.2017.01.013 28126693

[45] Werling K, Bianco NA, Raitor M, Stingel J, Hicks JL, Collins SH, et al. AddBiomechanics: automating model scaling, inverse kinematics, and inverse dynamics from human motion data through sequential optimization. PLoS One. 2023;18(11):e0295152. doi: 10.1371/journal.pone.0295152 38033114 PMC10688959

[46] Hammond CV, Williams ST, Vega MM, Ao D, Li G, Salati RM, et al. The neuromusculoskeletal modeling pipeline: MATLAB-based model personalization and treatment optimization functionality for OpenSim. bioRxiv. 2025:2024.10.30.620965. doi: 10.1101/2024.10.30.620965 40383769 PMC12087055

[47] Kolditz M, Albin T, Abel D, Fasse A, Brüggemann G-P, Albracht K. Simulative analysis of joint loading during leg press exercise for control applications. IFAC-PapersOnLine. 2015;48(20):435–40. doi: 10.1016/j.ifacol.2015.10.179

[48] Camomilla V, Cereatti A, Vannozzi G, Cappozzo A. An optimized protocol for hip joint centre determination using the functional method. J Biomech. 2006;39(6):1096–106. doi: 10.1016/j.jbiomech.2005.02.008 16549099

[49] Cereatti A, Della Croce U, Cappozzo A. Reconstruction of skeletal movement using skin markers: comparative assessment of bone pose estimators. J Neuroeng Rehabil. 2006;3:7. doi: 10.1186/1743-0003-3-7 16556302 PMC1435905

[50] Taylor WR, Ehrig RM, Duda GN, Schell H, Seebeck P, Heller MO. On the influence of soft tissue coverage in the determination of bone kinematics using skin markers. J Orthop Res. 2005;23(4):726–34. doi: 10.1016/j.orthres.2005.02.006 16022983

[51] Taylor WR, Kornaropoulos EI, Duda GN, Kratzenstein S, Ehrig RM, Arampatzis A, et al. Repeatability and reproducibility of OSSCA, a functional approach for assessing the kinematics of the lower limb. Gait Posture. 2010;32(2):231–6. doi: 10.1016/j.gaitpost.2010.05.005 20547061

[52] Zatsiorsky VM. Kinematics of human motion. Human Kinetics. 1998.

[53] Hamill J, Selbie WS, Kepple TM. Three-dimensional kinematics. Research methods in biomechanics. 2nd edn. Champaign, Ill.: Human Kinetics. 2014.

[54] Selbie WS, Hamill J, Kepple TM. Three-dimensional kinetics. Research methods in biomechanics. 2nd edn. Champaign, Ill.: Human Kinetics. 2014. p. 151–76.

[55] Delp SL, Loan JP, Hoy MG, Zajac FE, Topp EL, Rosen JM. An interactive graphics-based model of the lower extremity to study orthopaedic surgical procedures. IEEE Trans Biomed Eng. 1990;37(8):757–67. doi: 10.1109/10.102791 2210784

[56] Yamaguchi GT, Zajac FE. A planar model of the knee joint to characterize the knee extensor mechanism. J Biomech. 1989;22(1):1–10. doi: 10.1016/0021-9290(89)90179-6 2914967

[57] Meireles S, De Groote F, Reeves ND, Verschueren S, Maganaris C, Luyten F, et al. Knee contact forces are not altered in early knee osteoarthritis. Gait Posture. 2016;45:115–20. doi: 10.1016/j.gaitpost.2016.01.016 26979892

[58] Seth A, Sherman M, Reinbolt JA, Delp SL. OpenSim: a musculoskeletal modeling and simulation framework for in silico investigations and exchange. Procedia IUTAM. 2011;2:212–32. doi: 10.1016/j.piutam.2011.04.021 25893160 PMC4397580

[59] Stemmler M. Iterative learning control of medical applications. Germany: RWTH Aachen. 2023. https://publications.rwth-aachen.de/record/971460

[60] Martin Bland J, Altman D. Statistical methods for assessing agreement between two methods of clinical measurement. The Lancet. 1986;327(8476):307–10. doi: 10.1016/s0140-6736(86)90837-8 2868172

[61] Aristidou A, Cameron J, Lasenby J. Predicting missing markers to drive real-time centre of rotation estimation. Lecture Notes in Computer Science. Berlin, Heidelberg: Springer; 2008. p. 238–47. doi: 10.1007/978-3-540-70517-8_23

[62] Aristidou A, Cameron J, Lasenby J. Real-time estimation of missing markers in human motion capture. In: IEEE Proceedings of the 2nd International Conference on Bioinformatics and Biomedical Engineering. Shanghai, China: IEEE; 2008. p. 1343–6

[63] Keizer MNJ, Otten E. Technical note: sensitivity analysis of the SCoRE and SARA methods for determining rotational axes during tibiofemoral movements using optical motion capture. J Exp Orthop. 2020;7(1):6. doi: 10.1186/s40634-020-0219-z 32040787 PMC7010897

[64] Ketelhut M, Goll F, Braunstein B, Albracht K, Abel D. Iterative learning control of an industrial robot for neuromuscular training. In: 2019 IEEE Conference on Control Technology and Applications (CCTA). 2019. p. 926–32. doi: 10.1109/ccta.2019.8920659

[65] Ketelhut M, Brügge GM, Göll F, Braunstein B, Albracht K, Abel D. Adaptive iterative learning control of an industrial robot during neuromuscular training. IFAC-PapersOnLine. 2020;53(2):16468–75. doi: 10.1016/j.ifacol.2020.12.741

[66] Ketelhut M, Göll F, Braunstein B, Albracht K, Abel D. Comparison of different training algorithms for the leg extension training with an industrial robot. Curr Direct Biomed Eng. 2018;4(1):17–20. doi: 10.1515/cdbme-2018-0005

[67] Ketelhut M, Kolditz M, Göll F, Braunstein B, Albracht K, Abel D. Admittance control of an industrial robot during resistance training. IFAC-PapersOnLine. 2019;52(19):223–8. doi: 10.1016/j.ifacol.2019.12.102

[68] Schuengel V, Braunstein B, Goell F, Braun D, Reißner N, Safronov K, et al. Integrative biomechanics of a human–robot carrying task: implications for future collaborative work. Auton Robot. 2025;49(1). doi: 10.1007/s10514-024-10184-2

[69] Lloyd D. The future of in-field sports biomechanics: wearables plus modelling compute real-time in vivo tissue loading to prevent and repair musculoskeletal injuries. Sports Biomech. 2024;23(10):1284–312. doi: 10.1080/14763141.2021.1959947 34496728

