# Supplementary material for: Advancing knee adduction moment prediction for neuromuscular training via functional joint definitions and real–time simulation using OpenSim
Source: PLoS One. 2025 Jun 10;20(6):e0324985. doi: 10.1371/journal.pone.0324985 (PMC12151370; doi:10.1371/journal.pone.0324985)
Supplement: S1 Table — (PDF) [file pone.0324985.s001.pdf]

Table S1. Markers at anatomical landmark and in clusters on the torso, pelvis and the right extremity segments.

Segment	Anatomical landmark
Foot	Metatarsal head II
	Side of metatarsal head I
	Side of metatarsal head V
	Tip of toe II
	Heel
	Medial side on calcaneus
	Lateral side on calcaneus
Shank	Medial malleolus
	Lateral malleolus
	Four–point cluster (dorsolateral)
	Caput fibulae
	Tuberositas tibiae
Thigh	Medial epicondyle
	Lateral epicondyle
	Four–point cluster (dorsolateral)
Pelvis	Anterior superior iliac spine
	Posterior superior iliac spine
	Most proximal iliac crest
Torso	12th thoracic vertebra
	7th cervical vertebra
	Incisura jugolaris
	Processus Xyphoideus
	Four–point cluster mid chest
