# Supplementary material for: Advancing knee adduction moment prediction for neuromuscular training via functional joint definitions and real–time simulation using OpenSim
Source: PLoS One. 2025 Jun 10;20(6):e0324985. doi: 10.1371/journal.pone.0324985 (PMC12151370; doi:10.1371/journal.pone.0324985)
Supplement: S1 Fig — C3d files containing 3D marker trajectories and force data (green) are pre-processed. The generic OpenSim model is scaled according to the individualized model variations which is described in detail in Sects 2.9 and 2.10 and displayed in Fig 3A. (PDF) [file pone.0324985.s002.pdf]

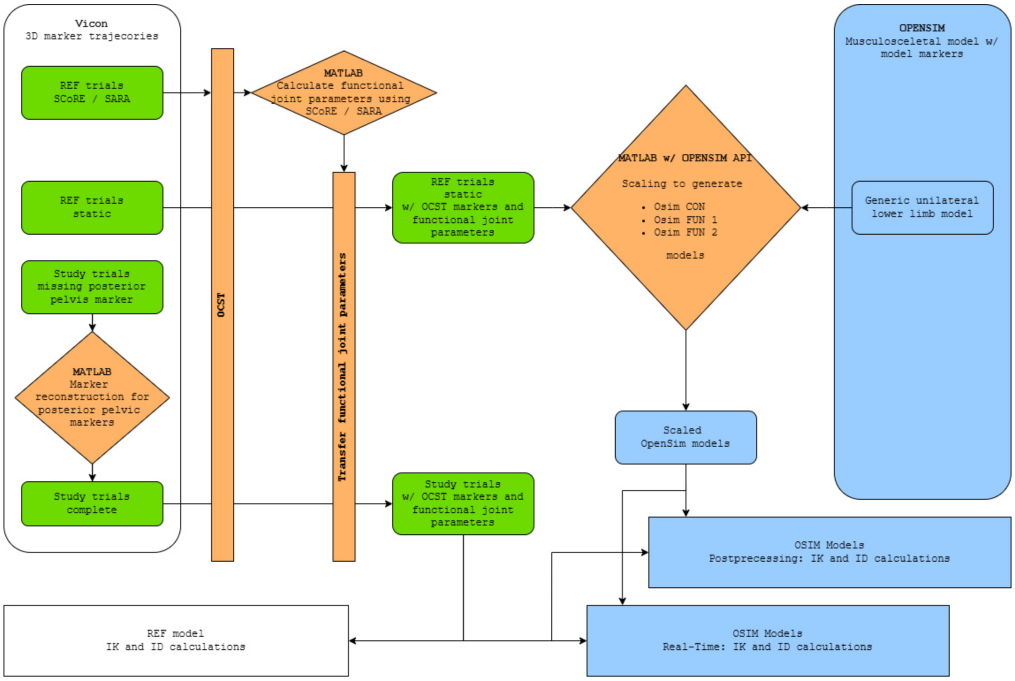

S1 Fig. Workflow schematic of the overall pre- and post-processing approach. C3d files containing 3D marker trajectories and force data (green) are pre-processed using OCST, SCoRE and SARA and marker transfer methods. The generic OpenSim model is scaled according to the individualized model variations used (blue) which is described in detail in sections 2.9 and 2.10 and displayed in Fig. 3A.
