# Supplementary material for: Advancing knee adduction moment prediction for neuromuscular training via functional joint definitions and real–time simulation using OpenSim
Source: PLoS One. 2025 Jun 10;20(6):e0324985. doi: 10.1371/journal.pone.0324985 (PMC12151370; doi:10.1371/journal.pone.0324985)
Supplement: S2 Fig — (PDF) [file pone.0324985.s003.pdf]

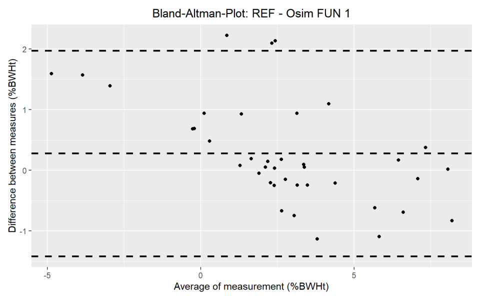

(a)

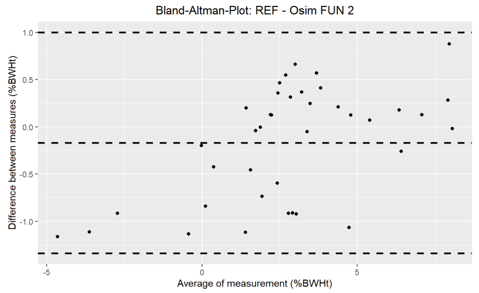

(b)

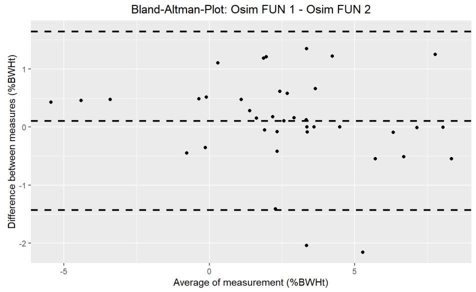

(c)

S2 Fig. Bland–Altman plot for the comparison of the external knee adduction moment (EAM) calculated from the static trials with the REF and the Osim FUN 1 model (a), calculated with the REF and the Osim FUN 2 model (b), and calculated with the Osim FUN 1 and the Osim FUN 2 model.
