# Supplementary material for: Advancing knee adduction moment prediction for neuromuscular training via functional joint definitions and real–time simulation using OpenSim
Source: PLoS One. 2025 Jun 10;20(6):e0324985. doi: 10.1371/journal.pone.0324985 (PMC12151370; doi:10.1371/journal.pone.0324985)
Supplement: S3 Fig — (PDF) [file pone.0324985.s004.pdf]

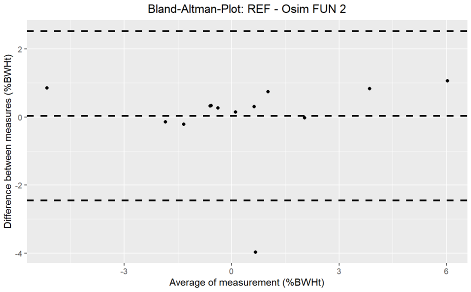

(a)

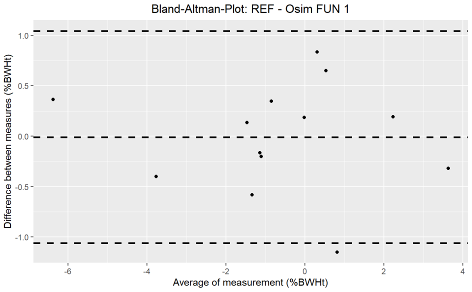

(b)

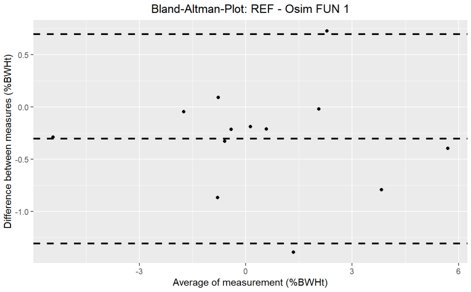

(c)

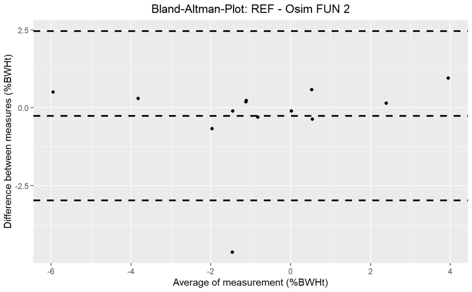

(d)

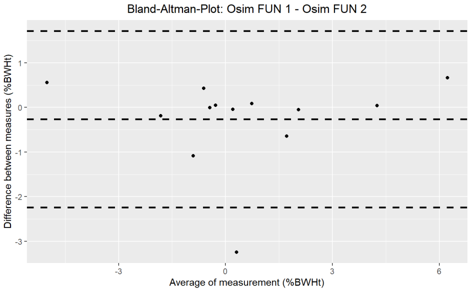

(e)

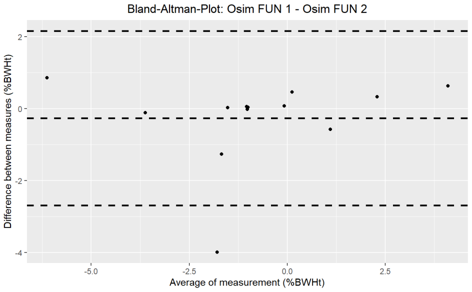

(f)

S3 Fig. Bland–Altman plot for the comparison of the mean external knee adduction moment (EAM) calculated from the dynamic trials with the REF and the Osim FUN 1 model (a), calculated with the REF and the Osim FUN 2 model (c), and calculated with the Osim FUN 1 and the Osim FUN 2 model (d) and for the comparison of the maximum external knee adduction moment (EAM) calculated from the dynamic trials with the REF and the Osim FUN 1 model (b), calculated with the REF and the Osim FUN 2 model (d), and calculated with the Osim FUN 1 and the Osim FUN 2 model (f).
