# Supplementary material for: Advancing knee adduction moment prediction for neuromuscular training via functional joint definitions and real–time simulation using OpenSim
Source: PLoS One. 2025 Jun 10;20(6):e0324985. doi: 10.1371/journal.pone.0324985 (PMC12151370; doi:10.1371/journal.pone.0324985)
Supplement: S4 Fig — (PDF) [file pone.0324985.s005.pdf]

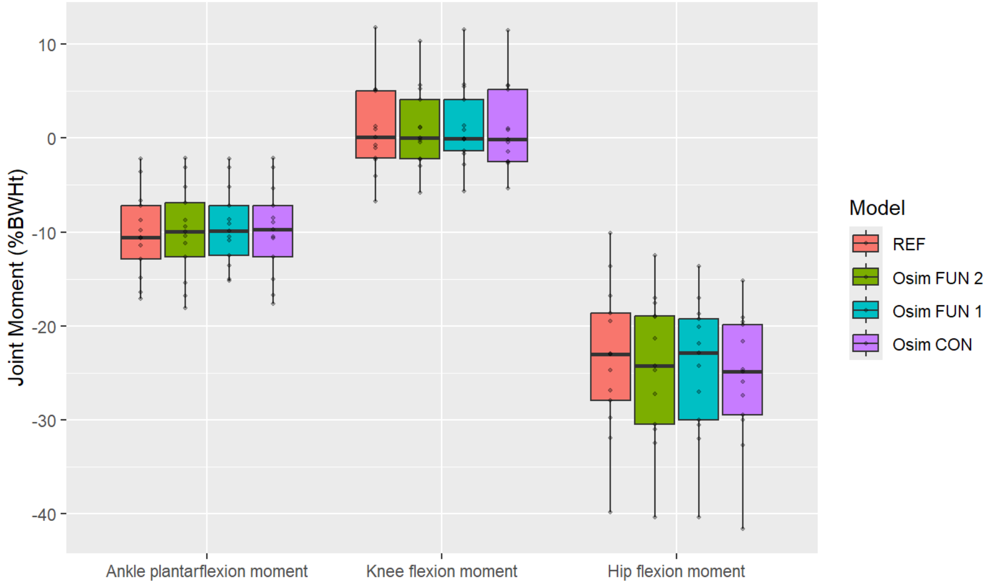

S4 Fig. Sagittal joint moment for dynamic trials
Boxplots representing the maximum ankle joint dorsalfalexion, knee joint flexion and the hip joint flexion momdent of the dynamic legpress trials calculated with the REF model (red), the Osim CON model (purple), the Osim FUN 2 model (green), and the Osim FUN 1 model (blue).
